# A common-sense model–based nursing intervention improves exercise compliance in coronary heart disease: a randomized controlled trial and a pilot study

**DOI:** 10.3389/fcvm.2025.1579015

**Published:** 2025-08-08

**Authors:** Jin Wang, Ying Zhou, Wanxu Huang, Huan Yin, Xiaofang Zhu, Zhenshuai Yao, Bo Dong, Pingping He

**Affiliations:** ^1^ Aging Health Research Center, School of Nursing, Health Science Center, Hunan Normal University, Changsha, China; ^2^ Hunan Provincial People's Hospital, The First-Affiliated Hospital of Hunan Normal University, Changsha, Hunan, China

**Keywords:** randomized controlled trial, coronary heart disease, common-sense model of self-regulation, exercise rehabilitation compliance, health outcomes, nursing intervention

## Abstract

**Aims:**

This study explored the effects of a Common-sense model of self-regulation-based nursing intervention on enhancing cardiac exercise rehabilitation compliance in coronary heart disease patients, aiming to improve cardiac function and overall health outcomes.

**Design:**

This study was a two-arm, parallel prospective randomized controlled trial.

**Methods:**

Participants were recruited from February to August 2024 at 3 Hospitals in Changsha, Hunan, China. Participants in the intervention group received a nursing intervention based on a Common-sense model of self-regulation and routine health education, while those in the control group received routine health education only. The outcome variables included exercise compliance, level of exercise fear, brief illness perception, emotional regulation self-efficacy level, blood pressure, body mass index, six-minute walking test. Statistical methods used to analyze the data include t-test, non-parametric rank sum test.

**Results:**

77 participants completed the study. Compared to the control group (*n* = 38), the intervention group (*n* = 39) showed statistically significant improvements in the outcomes of exercise compliance, level of exercise fear, level of brief illness perception, level of emotion regulation self-efficacy, blood pressure, body mass index, six-minute walk test.

**Conclusion:**

A Common-sense model-based cardiac exercise rehabilitation compliance intervention effectively improves health outcomes in coronary heart disease patients and can be integrated into nursing practice to enhance clinical care.

**Clinical Trial Registration:**

identifier ChiCTR2400084280.

## 1 Introduction

Amid societal and economic progress, cardiovascular diseases (CVDs) have become a critical global health challenge, with prevalence showing a persistent upward trend (1). The World Health Organization (WHO) estimates that CVD-related deaths will surpass 25.5 million by the mid-21st century, with coronary heart disease (CHD) accounting for the majority of cases (2). CHD, a prevalent cardiac condition primarily resulting from cardiac dysfunction and organic lesions leading to insufficient myocardial blood supply, is characterized by high mortality and disability rates. Consequently, it imposes significant psychological and economic burdens on both affected individuals and their families (3).

Contemporary medical advancements facilitate prompt coronary revascularization, effectively restoring myocardial perfusion. Nevertheless, although these interventions confer short-term survival advantages, they fail to address the underlying risk factors for CHD, resulting in substantial long-term mortality (4). Some studies have shown that lifestyle modifications and risk factor management can significantly reduce the incidence of cardiovascular events and long-term mortality (5, 6). Consequently, the therapeutic focus has increasingly transitioned from acute-phase intervention and rescue to a comprehensive approach encompassing primary prevention and post-event rehabilitation (7). Exercise training constitutes a core component of contemporary cardiac rehabilitation (CR) programs (8). Exercise-centered cardiac rehabilitation has gained established consensus as a key strategy for both CHD prevention and post-event rehabilitation. Major cardiovascular societies, including the European Society of Cardiology, the American Heart Association and the American College of Cardiology all recommending exercise rehabilitation for patients with CHD based on the highest level of scientific evidence (Level I) (9, 10). Recent systematic reviews confirm both the safety and long-term efficacy of remote cardiac rehabilitation in coronary populations, underlining the urgency to develop personalized and scalable models (11).

Nursing interventions employing the Common-sense model of self-regulation（CSM）as a theoretical framework have demonstrated efficacy in enhancing self-regulatory behaviors and relevant health outcomes among individuals with various chronic diseases (12, 13). However, there is no such intervention to improve compliance to cardiac exercise rehabilitation and health outcomes in patients with CHD.

## 2 Background

Research demonstrates that exercise-based cardiac rehabilitation constitutes an effective secondary prevention strategy for CHD, improving cardiac rhythm, blood flow, blood pressure, and lipid profiles (14). Cardiac exercise rehabilitation controls cardiovascular disease risk factors and improves cardiac and coronary artery function. Furthermore, compared to non-exercise control groups, this intervention significantly reduces the risk of cardiovascular mortality and hospitalization, while improving health-related quality of life (15). Additionally, it promotes mental health by providing patients with a platform to address concerns, effectively alleviating anxiety (16).

Cardiac exercise rehabilitation compliance refers to the extent to which patients follow healthcare professionals' recommendations, including participation in rehabilitation programs, exercise training, dietary guidelines, medication regimens, and other prescribed behaviors (17). Exercise rehabilitation compliance is essential for achieving optimal therapeutic outcomes in CHD patients, yet their adherence rates are generally low (18). I Factors such as age, literacy, disease awareness, coping styles, psychological status, and disease-related uncertainty negatively impact CHD exercise rehabilitation compliance (19). Evidence indicates that diminished patient perception of disease symptoms and effects corresponds to reduced treatment compliance (20). Additionally, fear of exercise, commonly observed in CHD patients, substantially reduces cardiac rehabilitation participation, stemming from fears of precipitating cardiac events (21). Negative emotions and entrenched lifestyle habits among coronary artery disease patients frequently compromise long-term exercise compliance, however, support from peers and family can enhance participation motivation (22). Therefore, altering level of illness perception, level of exercise fear, and emotion regulation in patients with coronary artery disease can promote compliance to cardiac exercise rehabilitation.

**Figure 1:**
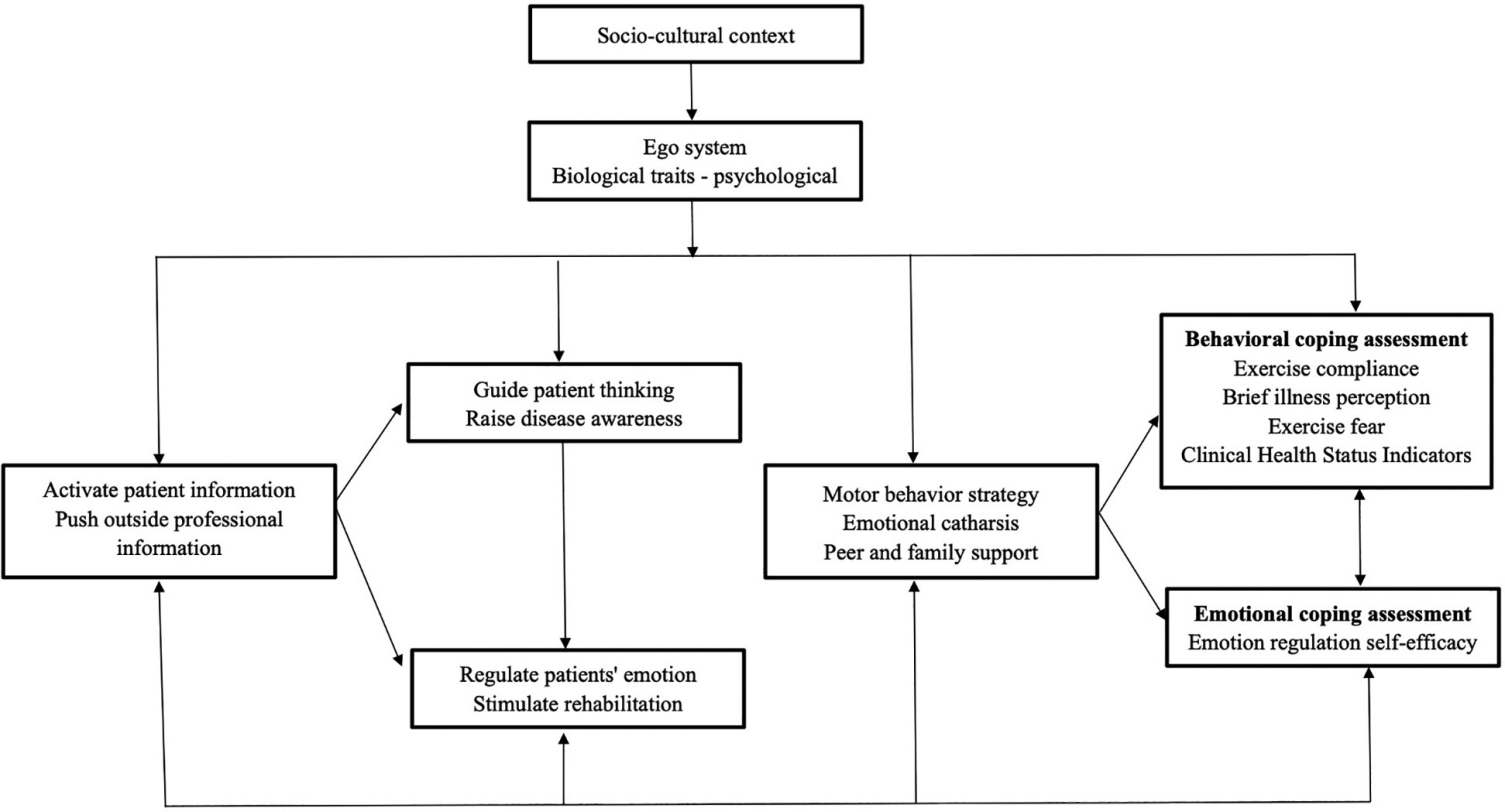
Application research framework of intervention based on CSM in compliance with cardiac exercise rehabilitation in patients with CHD.

The CSM provides a theoretical framework for examining the relationship between an individual's illness perception, coping, and health outcomes in the aftermath of an illness (Figure 1) (23). The CSM posits that individuals take action to mitigate health risks based on their subjective understanding and commonsense perceptions of health threats, providing a comprehensive framework for understanding the relationships between health threats, coping responses, and disease outcomes. The model comprises four interconnected components: (1) health threats, which encompass both internal indicators (e.g., physical symptoms) and external information sources (e.g., electronic media); (2) illness representation, consisting of cognitive and emotional dimensions that characterize health threats; (3) coping procedures, representing self-regulatory strategies developed in response to illness representations; and (4) outcome assessment, involving continuous evaluation of coping effectiveness throughout disease management. Compared to other health education models, CSM offers unique intervention targets across three phases (cognition, response, and assessment), leveraging both intrinsic and extrinsic motivation to enhance health beliefs and improve exercise rehabilitation adherence (24, 25).

Evidence demonstrates that interventions grounded in the Common-Sense Model of Self-Regulation facilitate the development of healthy behaviors and effective self-management practices to optimize health outcomes (26, 27), thereby improving their healthcare behaviors, compliance, quality of life, and alleviating their anxiety, depression and psychological distress (26, 28).

However, CSM is rarely used in health interventions for patients with CHD. Therefore, we undertook to address this gap by developing a CSM-based nursing intervention to improve the impact of compliance to cardiac exercise rehabilitation and health outcomes in Chinese CHD.

## 3 Methods

### 3.1 Study design

A two-arm, parallel prospective randomized controlled trial design was employed.

### 3.2 Aims

Aims of this study are (1) to develop a nursing intervention based on CSM and (2) to explore whether the intervention could improve exercise rehabilitation compliance and health outcomes.

### 3.3 Participants

Participants were recruited between February and August 2024 from 3 hospitals in Changsha, Hunan, China. Based on the study objectives, ethical considerations, the voluntary nature of participation, and required physical fitness levels, we established the following inclusion and exclusion criteria.

Individuals were included if they:

(1) Age over 18 years old, both genders were included
(2) Patients with CHD who meet the WHO diagnostic criteria for CHD
(3) Clearly conscious and have certain language expression ability
(4) Able to use WeChat simply or under guidance, and willing to accept WeChat or telephone follow-up
(5) NYHA classified cardiac function grade Ⅰ∼Ⅲ
(6) Patients gave informed consent and voluntarily participated in this study

Individuals were excluded if they:

(1) People with severe hearing and vision impairment
(2) Those with mental abnormalities and cognitive disorders
(3) Patients with other serious diseases in combination
(4) Patients with motor dysfunction or physical disability
(5) Patients with contraindications to exercise rehabilitation, such as unstable angina pectoris, acute myocardial infarction, uncontrolled cardiac arrhythmia, blood pressure > 220 mmHg, malignant tumor, and so on

Individuals were rejected if they:

(1) Transferred to another hospital or aggravated during the study
(2) Withdrawal from the study or loss of contact due to personal reasons on their own initiative

The main observable in this study was exercise rehabilitation compliance, based on the two-sample mean comparison sample size formula: $n_{1}=n_{2}=2{\left[\frac{\left(\mu_{\alpha }+\mu_{\beta }\right)}{\delta /\sigma }\right]}^{2}+\frac{1}{4}\mu_{\alpha }^{2}\;$, Where *μ_α_* and μ*_β_* are the μ values corresponding to the probability of Type I error and Type II error, respectively, *σ* is the estimated value of the standard deviation of the two totals, which is generally taken as the greater of the two, and *δ* is the difference between the two means, and in this study, it is assumed that *α* = 0.05 and *β* = 0.1 to do a two-sided test. Checking the table, μ*_α_* = μ_0.05/2_ = 1.96, μ*_β_* = μ_0.1_ = 1.282, *δ*/*σ* is 0.84, substituting the data into the formula calculates n1 = n2 = 31, considering the 20% loss of visit rate, the sample size was finally determined by calculation to be 80 cases, with 40 cases in each of the control group and the intervention group.

**Figure 2:**
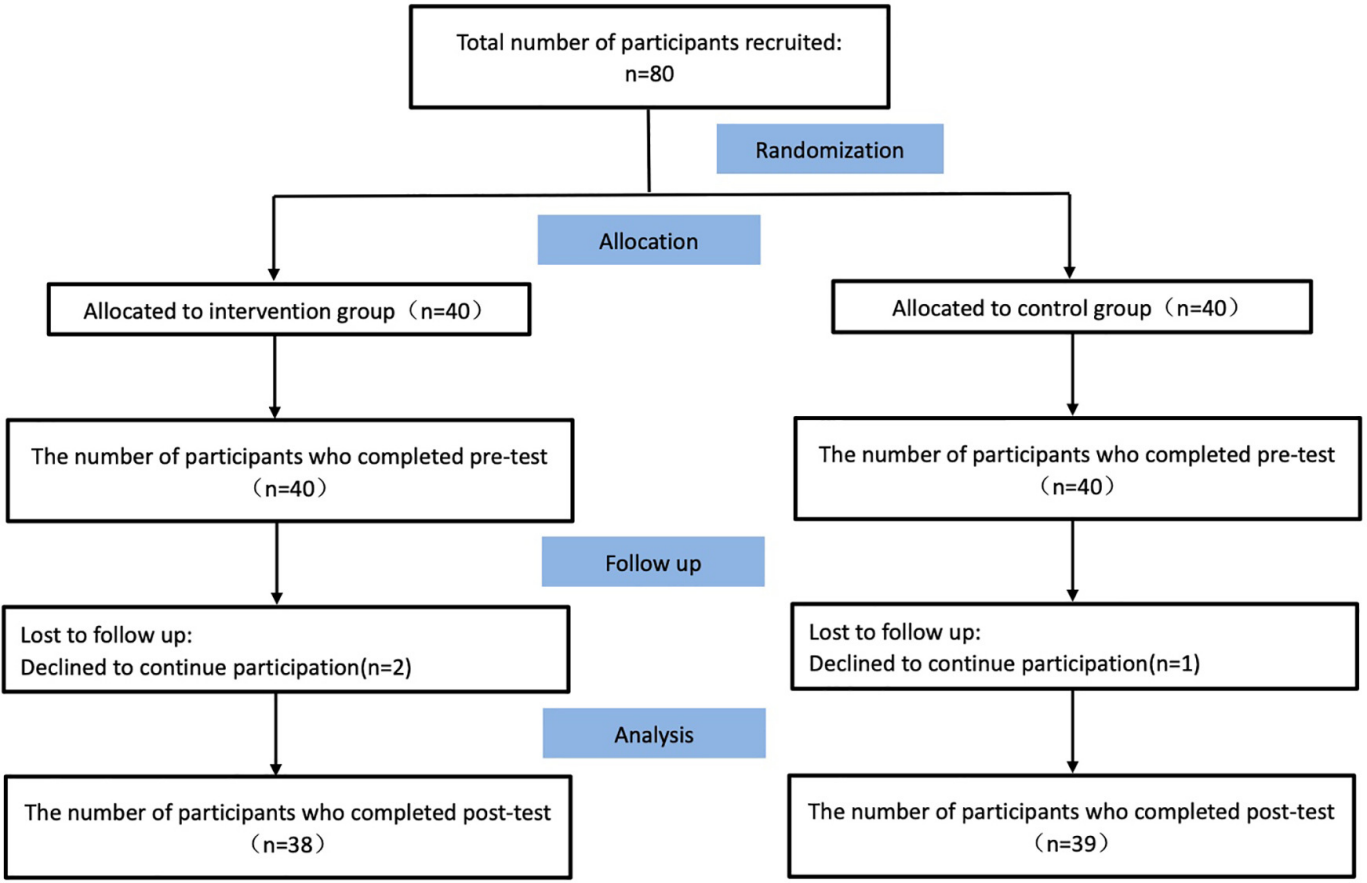
CONSORT flow diagram.

This two-arm, parallel, prospective randomized controlled trial recruited eligible participants prior to randomization to mitigate recruitment bias. A random number sequence generated in Excel was used to allocate patients sequentially to either the intervention or control group. During the intervention, 2 participants in the intervention group were lost (1 due to disease progression, unrelated to exercise rehabilitation; 1 due to work commitments), resulting in a 5.26% attrition rate. One control group participant was lost due to changed contact information (2.63% attrition). The final analysis included 77 participants: 38 in the intervention group and 39 in the control group (Figure 2).

### 3.4 The routine health education

Routine in-hospital health education, including the provision of CHD health education, cardiac exercise rehabilitation manuals and other materials; after discharge from the hospital, the researcher conducted a telephone follow-up, once a month, a total of three times, during which compliance to cardiac rehabilitation and any existing primary issues were assessed.

### 3.5 The nursing intervention

The intervention group received the same routine health education plus a nursing intervention based on the Common-sense model of self-regulation.

#### 3.5.1 Building an intervention team

A multidisciplinary team with clearly delineated responsibilities was established. A doctoral supervisor oversaw intervention implementation, quality control, and study coordination. A cardiology chief nurse (associate chief superintendent nurse) coordinated staffing for the intervention components. Two cardiologists delivered training sessions and lectures. A rehabilitation therapist (associate rehabilitation therapist) guided patients' early-stage rehabilitation exercise. A psychotherapist managed patients' negative emotion. Two cardiology nurses conducted health education and assisted with post-discharge follow-up. Two nursing postgraduate students performed data collection and statistical analysis.

#### 3.5.2 The intervention time

The 12-week intervention protocol, structured into three distinct phases, was designed in accordance with the Chinese Expert Consensus on Cardiac Rehabilitation and Secondary Prevention for Coronary Heart Disease. This duration aligns with standard clinical practice in China, where CHD patients typically undergo follow-up assessments at the 3-month mark (approximately 12 weeks).

Phase 1 (Week 1): Corresponds to the average hospitalization period for CHD patients, during which in-hospital rehabilitation guidance will be delivered.

Phase 2 (Weeks 2–8): Implements the post-discharge exercise rehabilitation program prior to the 3-month follow-up.

Phase 3 (Week 9–12): Phase 3 was timed to coincide with the 3-month follow-up assessment.

#### 3.5.3 The intervention site

The intervention sites were categorized as in-hospital and out-of-hospital. The in-hospital sites comprised the cardiovascular medicine wards and the cardiac rehabilitation center, while out-of-hospital intervention was delivered remotely, primarily via WeChat groups and telephone follow-up.

#### 3.5.4 Content of the intervention

The intervention program as shown in Table 1.

**Table 1:** Intervention program of cardiac exercise rehabilitation compliance based on common sense model for patients with CHD.

Subject	Theme	Content	manner	Time and place
Stimulus of health threats	Inspire healthy beliefs and build trusting relationships	1. Internal Stimulation: Activate Patients’ Information (1) Investigate patients’ understanding about CHD. (2) Investigate patients’ understanding of cardiac rehabilitation exercise. 2. External Stimulus: Provide External Professional Information (1) Conducted lectures and distributed educational manuals introducing the definition of CHD, management of cardiovascular disease risk factors, specific contents of the five major prescriptions for cardiac rehabilitation, and knowledge of cardiac exercise rehabilitation. (2) Establish WeChat groups to targetedly push external professional information based on patients’ specific conditions, to meet the actual needs of different patients.	Group intervention + individual intervention (1) 10 min/person at the bedside; (2) Special lectures: 30min-45 min; (3) Establish a WeChat communication group; (4) Push the official account related to cardiac rehabilitation	Inside the hospital; (Phase I); 1st week 1st; Cardiology ward, propaganda classroom
Reconstruction of the disease cognition table	Guide patients to think and improve disease awareness	1. Assess cognitive representations of disease (1) Subjective: Understand the patient's subjective view of the disease and cardiac rehabilitation exercise. (2) Objective: The investigator instructs the patient to fill in the simplified version of the disease perception questionnaire. 2. In-depth understanding of illness and recovery (1) Introduce the time node of the third phase of cardiac rehabilitation; (2) Introduce the importance and scientificity of insisting on cardiac exercise rehabilitation; (3) Use real and typical examples to emphasize that CHD is seriously affected by bad behaviors and encourage patients to actively participate in rehabilitation training.	Group intervention + individual intervention (1) 10 min/person at the bedside; (2) Special lectures: 30min-45 min; (3) The official account in the WeChat group is pushed 1 time	In-hospital; (Phase I); 2nd time in week 1; Cardiology ward, propaganda classroom
Assist in catharsis	Regulate patients’ emotions and stimulate their motivation for recovery	1. Assess mood representations of the disease (1) Subjective: assess the patient's emotional state and distress; (2) Objective: The investigator instructs the patient to fill in the Coronary Heart Disease Fear of Movement Scale. 2. Patients are encouraged to express themselves (1) Strengthen daily emotional communication with patients, encourage them to voice their difficulties, and adopt methods such as explanation, support, and encouragement to alleviate and eliminate negative emotions; emphasize the benefits of actively participating in exercise behavior to patients. (2) Share experiences and summarize lessons: Before discharge, encourage patients to summarize their “exercise rehabilitation experience” in writing or orally, highlighting the benefits gained and obstacles overcome; recruit patients who have benefited significantly from previous exercise rehabilitation participation and utilize online platforms for them to share their experiences.	Individual interventions (1) 10 min/person at the bedside; (2) Experience sharing in WeChat group for 30 min	Inside the hospital; (Phase I); 3rd week 1; Cardiology ward
Coping strategy development	1.Evaluation and goal setting	1. Pre-discharge assessment: Evaluate the patient's medical history, cardiovascular function, and exercise environment in detail, and conduct a detailed physical examination. 2. Exercise goal setting (1) Develop personalized exercise prescription: follow the FITT-VP principle, that is, the principle of exercise frequency, intensity, form, time, exercise amount, and progressiveness; (2) Formulate personalized exercise intensity: the self-perceived exertion degree grading method combined with the heart rate reserve method was used to formulate the exercise intensity according to the risk stratification;	Individual interventions (1) One-on-one exercise assessment for 1 time; (2) Set an exercise goal 1 time	Inside the hospital; (Phase I); Week 2; Cardiac Rehabilitation Center
	2. Exercise guidance and execution	1. In-hospital exercise rehabilitation guidance: Personalized exercise guidance is formulated for patients: exercise in bed, bedside sitting, bedside standing, and bedside activities; 2. Out-of-hospital exercise rehabilitation guidance Step 1: Warm-up activities; Step 2:The rehabilitation therapist recommends exercise patterns, aerobic exercise and other exercises according to the patient's risk level; Step 3: Relaxation exercises.	Group intervention + individual intervention (1) Distribution of sports education manuals; (2) Demonstration of the essentials of exercise rehabilitation for 1 time; (3) Push the exercise training teaching video 1 time; (4) Telephone guidance for patients to exercise 1 time/week, a total of 4 times	Outside the hospital; (Stage II); Week 3 - Week 8; Cardiac Rehabilitation Center/Home (Online).
	3. Health education and safe exercise	1. Exercise monitoring (1) The researchers instructed the patients to use electronic exercise bracelets and WeChat exercises to record their movements; (2) the patient's completion of exercise rehabilitation log; (3) In the fourth week, the rehabilitation therapist instructed the researchers to evaluate the effect of exercise on the patient, and dynamically adjusted the exercise prescription according to the rehabilitation situation. 2. Health education (1) Low-fat diet (2) Smoking cessation guidance (3) Keep the stool smooth, avoid mental tension and emotional agitation, and avoid strenuous physical labor. (4) Ensure good sleep quality. (5) Take your medication on time.	Group intervention + individual intervention (1) WeChat group exercise check-in (upload video/photo) 3 times/week (2) Distribution of health education brochures	
Outcome assessment	Assess patient behavior and mood, mitigate health threats	1. WeChat and Telephone Follow-up: (1) Rehabilitation therapists weekly direct researchers to post sports rehab content in WeChat groups. (2) Docs & nurses weekly collect & sort patient feedback, addressing individual queries one-on-one. (3) Researchers weekly guide patients via WeChat/phone on rehab progress & exercise adherence. 2. Outpatient Follow-up: (1) Emotional Outcome Assessment: Use the self-efficacy scale to evaluate patients’ emotions at Week 12. (2) Exercise Effect Evaluation: Collect logs, questionnaires on compliance, disease perception, & motor fear; reward high compliance with gifts; support low-compliance patients.	Group intervention + individual intervention (1) outpatient follow-up (week 12); (2) WeChat and telephone follow-up visits 3 times/week, a total of 12 times	Outside the hospital; (Stage II); Week 9 - Week 12; Cardiac Rehabilitation Center/Home (Online).

### 3.6 Outcome measures

In accordance with the aim of our study, we collected demographic data, the Exercise Compliance Questionnaire, the Fear of Exercise Scale for Patients with Coronary Artery Disease, the Brief Illness Perception Questionnaire, the Emotion Regulation Self-Efficacy Scale.

#### 3.6.1 Exercise compliance questionnaire

The exercise compliance questionnaire was developed by XueLi (29), comprises three dimensions: physical exercise compliance, exercise monitoring compliance, and proactive advice-seeking compliance, totaling 12 items. A 4-point Likert scale was used, ranging from “Cannot do at all” (1) to “Can do completely” (4), with total scores ranging from 12 to 48. The Cronbach's alpha coefficient was 0.818.

#### 3.6.2 The fear of exercise scale for patients with coronary artery disease, fact-CAD

The Fear of Exercise Scale for Patients with Coronary Artery Disease (Fact-CAD) was translated into Chinese by Cuiping Tian (30). A total of 19 items were used. Likert 5-point scale was used, from “never” to “always” were assigned 0–4 points, and the total score was 0–76 points, and some of the entries were reverse scored, the higher the score, the higher the level of exercise fear of the patients. The Cronbach's alpha coefficient of this scale was 0.901.

#### 3.6.3 Brief illness perception questionnaire, BIPQ

The Simple Illness Perception Questionnaire (BIPQ) was developed by Broadbent (31), assesses cognitive, emotional, and understanding dimensions of illness perception. It includes 8 items scored on an 11-point scale and 1 open-ended question for listing perceived causes of the illness. Items 3, 4, and 7 are reverse-scored, with total scores (sum of items 1–8) indicating stronger perceived symptom severity and disease impact. The scale demonstrated a Cronbach's alpha of 0.831 and test-retest reliability of 0.931.

#### 3.6.4 Emotion regulation self-efficacy scale

The Emotion Regulation Self-Efficacy Scale (ERSES) was developed by Caprara (32) and later translated by Shufeng Wen (33) to measure the degree to which patients with CHD feel confident in their ability to manage their emotions. The scale consists of 12 items, including self-efficacy for expressing positive emotions (4 items), self-efficacy for regulating anger/rage (4 items), and self-efficacy for regulating frustration/pain (4 items), and is rated on a 5-point scale from “very poorly” to “very well”. The scale is rated on a 5-point scale from “very unconformable” to “very conformable”, with scores ranging from 1 to 5 and 12 to 60, with higher scores indicating higher levels of self-management of emotions. The Cronbach's coefficient of the scale was 0.85, and the factor model fit was good.

### 3.7 Clinical health Status indicators

① Blood pressure: Patients whose blood pressure meets the standard (<65 years old, blood pressure <140/90 mmHg; ≥65 years old, blood pressure <150/90 mmHg). Patients whose blood pressure is not up to standard (<65 years old, blood pressure ≥140/90 mmHg; ≥ 65 years old, blood pressure ≥150/90 mmHg).

② Body Mass Index (BMI): BMI = weight (kg)/height 2 (m^2^), normal: 20.0–23.9 kg/m^2^; overweight: BMI 24.0–27.9 kg/m^2^; obese: BMI ≥ 28.0 kg/m^2^.

③ Six-minute walking test (6MWT): evaluate the exercise endurance through 6-minute walking distance of patients to determine the cardiac function and prognosis. The 6MWT can be divided into 4 levels, level 1 < 300 m, level 2 is 300–374.9 m, level 3 is 375–449.9 m, and level 4 is ≥450 m. The higher the level, the better the cardiac function, and level 3 or 4 indicates that the cardiac function is close to or has reached the normal level.

### 3.8 Procedure and data collection

Data were collected three times: at baseline (1 week before the intervention) and at follow-up (weeks 4 and 12 after the intervention).

Data were collected in participants' homes or hospital. Participants completed questionnaires by themselves or with some assistance from a researcher. It took about 30–40 min to complete the questionnaires. Data on clinical indicators were collected by hospital nurses.

### 3.9 Statistical analysis

SPSS 27.0 software was used for statistical description and analysis of this study. The statistical methods are as follows:

Comparison of patients “general data before intervention: the measured data obeys normal distribution with chi-square and is described by mean ± standard deviation, otherwise it is described by median and interquartile spacing; the count data in patients” general data is described by percentage.

Comparison of pre-intervention baseline data and post-intervention data at 2 time points: the data were quantitative data, and two independent samples t-test was used if the data conformed to normal distribution, and non-parametric rank-sum test was used for the quantitative data that did not obey normal distribution.

## 4 Results

### 4.1 Participants

Table 2 shows the self-reported characteristics of the 77 participants (42 males and 35 females) who completed the study. The mean age of the intervention group was 71 (67.8, 74.3) years and the mean age of the control group was 71 (65, 75) years. The largest proportion of participants for each characteristic were married (88.3%), had primary school education or less (66.2%), lived with family (88.3%), lived in urban areas (57.1%), retired (61.0%), had a household economic income >4000RMB (55.8%), had urban and rural health insurance (59.7%), had no usual exercise habits 62.3%), and stable coronary heart disease (61.0%), no experience of PCI treatment (81.8%), no experience of CABG (80.5%), duration of coronary artery disease >5 years (39.0%), 2 hospitalizations for coronary artery disease (51.9%), cardiac function class 2 (81.8%), and coronary artery disease in combination with 2 other chronic diseases (41.6%); Table 2). Differences between the two groups in all demographic and disease-related characteristics were not statistically significant (all *p* > 0.05).

**Table 2:** Demographic characteristics (*n* = 77).

Variables	Grouping	Intervention group （n = 38）	Control group （n = 39）	*χ^2^/Z*	*P*
Age（year）		71（67.8, 74.3）	71（65, 75）	0.034^a^	0.973
Gender	Male	22（57.89）	20（51.28）	0.339^b^	0.536
	Female	16（42.1）	19（48.72）		
Marital status	Married	35（92.11）	33（84.62）	1.246^b^	0.669
	Divorced	2（5.27）	3（7.69）		
	Widowed	1（2.62）	3（7.69）		
Highest education level	Primary school or less	24（63.16）	27（69.23）	1.082^b^	0.884
	Junior high school	9（23.68）	6（15.38）		
	High school and secondary school	3（7.89）	4（10.26）		
	undergraduate and college diploma	2（5.26）	2（5.13）		
Living arrangements	Living with family	35（92.11）	33（84.62）	1.046^b^	0.306
	Live alone	3（7.89）	6（15.38）		
Home location	Countryside	8（21.05）	8（20.51）	1.822^b^	0.402
	County seat	6（15.79）	11（28.21）		
	City	24（63.16）	20（51.28）		
Occupation	Peasant	8（21.05）	8（20.51）	2.173^b^	0.848
	Worker	2（5.26）	2（5.13）		
	Teacher	0（0）	1（2.56）		
	Functionary	2（5.26）	1（2.56）		
	Retirement (from work)	24（63.16）	23（58.97）		
	Something else	2（5.26）	4（10.26）		
Family monthly income (RMB)	<2000	7（18.42）	8（20.51）	0.737^b^	0.692
	2000∼4000	11（28.95）	8（20.51）		
	>4,000	20（52.63）	23（58.97）		
Medical insurance	Rural cooperative medical care	21（55.26）	25（64.1）	3.836^c^	0.452
	Municipal medical insurance	10（26.32）	4（10.26）		
	Provincial medical insurance	1（2.63）	1（2.56）		
	Self-funded	1（2.63）	2（5.13）		
	Other options	5（13.16）	7（17.95）		
Exercise routine	Yes	17（44.74）	12（30.77）	1.599^b^	0.206
	No	21（55.26）	27（69.23）		
Types of Coronary Heart Disease	Stabilize	25（65.79）	22（56.41）	1.446^c^	0.664
	Instability	9（23.68）	14（35.9）		
	Myocardial infarction	4（10.53）	3（7.69）		
Has experienced PCI treatment	Yes	9（23.68）	5（12.82）	1.527^b^	0.217
	No	29（76.32）	34（87.18）		
Have experienced CABG	Yes	8（21.05）	7（17.95）	0.118^b^	0.731
	No	30（78.95）	32（82.05）		
Duration of coronary heart disease	<1	14（36.84）	13（33.33）	0.224^b^	0.894
	1∼5	9（23.68）	11（28.21）		
	>5	15（39.47）	15（38.46）		
Number of hospitalizations for coronary heart disease	1	11（28.95）	12（30.77）	1.574^b^	0.455
	2	22（57.89）	18（46.15）		
	>3	5（13.16）	9（23.08）		
Cardiac Function Classification	2	32（84.21）	31（79.49）	0.289^b^	0.591
	3	6（15.79）	8（20.51）		
Combination of other chronic diseases	0	4（10.53）	5（12.82）	3.523^c^	0.304
	1	14（36.84）	7（17.95）		
	2	14（36.84）	18（46.15）		
	≥3	6（15.79）	9（23.08）		

^a^ *Z*-value.

^b^ *χ^2^*.

^c^ Fisher's exact probability method value.

### 4.2 The effect of the nursing intervention on participants' exercise compliance

At the pre-test, the difference between the total exercise compliance score and the scores of all dimensions was not statistically significant when comparing the control group and the intervention group [t = (−0.850) – (−0.375), all *p* > 0.05]. At posttest, the intervention group at T1 had significantly higher total exercise compliance scores and scores on all dimensions (except exercise monitoring compliance) than the control group, with total exercise compliance scores (t = 5.272, *p* < 0.001); physical exercise compliance (t = 4.612, *p* < 0.001); exercise monitoring compliance (Z = −1.396, *p* > 0.05); and active advice-seeking compliance (Z = −2.219, *p* < 0.05). The total exercise compliance score and all dimension scores (except exercise monitoring compliance) were significantly higher in the intervention group than in the control group at T2, with total exercise compliance (Z = −4,744, *p* < 0.001); physical exercise compliance (t = 5.597, *p* < 0.001); exercise monitoring compliance (t = 0.948, *p* > 0.05); active advice seeking compliance (Z = −2.253, *p* < 0.05); overall, the differences were statistically significant (*p* < 0.05) for all dimensions except exercise monitoring compliance at 1 month post-intervention and 3 months post-intervention (Table 3).

**Table 3:** Effects of the nursing intervention on exercise compliance.

Variables	Time period	Intervention group（*n* = 38)	Control group（*n* = 39)	*t/Z*	*P*
Total exercise compliance scores	T_0_	24.05 ± 3.35	24.72 ± 3.51	−0.850^a^	0.398
	T_1_	30.45 ± 2.85	26.62 ± 3.48	5.272^a^	<0.001
	T_2_	35（32, 36.25）	31（29, 33）	−4.744^b^	<0.001
Physical exercise compliance	T_0_	11.16 ± 2.44	11.46 ± 2.47	−0.542^a^	0.589
	T_1_	14.37 ± 2.17	11.9 ± 2.51	4.612^a^	<0.001
	T_2_	17.00 ± 2.04	14.38 ± 2.06	5.597^a^	<0.001
Exercise monitoring compliance	T_0_	6.08 ± 1.62	6.21 ± 1.32	−0.375^a^	0.708
	T_1_	8（7, 9）	6（7, 8）	−1.396^b^	0.163
	T_2_	8.66 ± 1.30	8.36 ± 1.46	0.948^a^	0.346
Active advice-seeking compliance	T_0_	6.82 ± 1.56	7.05 ± 1.43	−0.691^a^	0.492
	T_1_	8（7, 9）	8（7, 9）	−2.219^b^	0.033
	T_2_	9（8, 10）	8（7, 9）	−2.253^b^	0.024

T0 pre-intervention.

T1 1-month post-intervention.

T2 3-months post-intervention.

^a^ *t*-value.

^b^ *Z*-value.

### 4.3 The effect of the nursing intervention on participants' fear of exercise

At the pre-test, the difference between the total score fear of exercise and the scores of all dimensions was not statistically significant when comparing the control group and the intervention group [t = 1.002, Z = (−1.718) −(−0.477), all *p* > 0.05]. At posttest, the intervention group at T1 had significantly higher total exercise compliance scores and scores on all dimensions (except exercise avoidance) than the control group, with total exercise fear scores (t = −4.262, *p* < 0.001); danger perception (Z = −3.039, *p* < 0.05); exercise fear (Z = −3.308, *p* < 0.05); and exercise avoidance (t = −0.358, *p* > 0.05); dysfunction (Z = −2.114, *p* < 0.05). The intervention group in T_2_ had significantly higher total motor fear scores and scores on all dimensions than the control group, with total motor fear (Z = −6.464, *p* < 0.001); danger perception (Z = −4.761, *p* < 0.001); motor fear (Z = −4.56, *p* < 0.001); motor avoidance (Z = - 3.114, *p* < 0.05); and dysfunction (Z = −2.686, *p* < 0.05). Overall, except for the motor avoidance dimension at 1 month post-intervention, the differences were statistically significant (*p* < 0.05) at 1 month post-intervention and 3 months post-intervention for all other dimensions (Table 4).

**Table 4:** Effects of the nursing intervention on fear of exercise.

Variables	Time period	Intervention group（*n* = 38)	Control group（*n* = 39)	*t/Z*	*P*
Total exercise fear scores	T_0_	40.74 ± 2.30	40.15 ± 2.78	1.002^a^	0.319
	T_1_	36.11 ± 2.29	38.46 ± 2.55	−4.262^a^	<0.001
	T_2_	32（30.75, 33）	36（35, 38）	−6.464^b^	<0.001
Danger perception	T_0_	10（9, 11）	10（9, 11）	−0.477^b^	0.633
	T_1_	9（8, 9）	10（8, 11）	−3.039^b^	0.002
	T_2_	8（7, 8）	9（8, 10）	−4.761^b^	<0.001
Exercise fear	T_0_	8（7, 9）	9（7, 9）	−1.454^b^	0.146
	T_1_	7（7, 8）	8（8, 8）	−3.308^b^	0.001
	T_2_	6（6, 7）	7（7, 8）	−4.56^b^	<0.001
Exercise avoidance	T_0_	13（12, 15）	13（11, 14）	−1.718^b^	0.086
	T_1_	11.79 ± 1.613	11.92 ± 1.66	−0.358^a^	0.721
	T_2_	10（9, 12）	11（10, 13）	−3.114^b^	0.002
Dysfunction	T_0_	9（9, 10）	9（8, 10）	−1.235^b^	0.217
	T_1_	8（8, 9）	9（8, 9）	−2.114^b^	0.035
	T_2_	8（7, 8）	8（8, 9）	−2.686^b^	0.007

T0 pre-intervention.

T1 1-month post-intervention.

T2 3-months post-intervention.

^a^ *t*-value.

^b^ *Z*-value.

### 4.4 The effect of the nursing intervention on participants' brief illness perception

At the pre-test, the difference in BIPQ total scores and scores of all dimensions was not statistically significant when comparing the control and intervention groups [t = 1.353, Z = (−1.891)-(−0.202), all *p* > 0.05]. At posttest, the intervention group at T1 had significantly higher BIPQ total scores and scores on all dimensions (except duration) than the control group, with BIPQ total scores (t = −5.095, *p* < 0.001); outcome (t = −6.400, *p* < 0.001); duration (Z = −1.208, *p* > 0.05); and personal control (Z = −5.579, *p* < 0.001); Disease control (Z = −2.679, *p* < 0.05); Symptoms (Z = −6.17, *p* < 0.001); Attention (Z = −2.928, *p* < 0.05); Understanding (Z = −3.267, *p* < 0.05); and Mood (Z = −5.497, *p* < 0.001). The intervention group of T2 had a significantly higher BIPQ total score and scores on all dimensions (except duration, disease control) were significantly higher than those of the control group, BIPQ total score (t = −8.065, *p* < 0.001); outcome (Z = −6.799, *p* < 0.001); duration (Z = −1.338, *p* > 0.05); personal control (Z = −6.35, *p* < 0.001); disease control (Z = −1.421, *p* > 0.05); symptoms (Z = −6.752, *p* < 0.001); attention (Z = −3.957, *p* < 0.001); understanding (Z = −6.469, *p* < 0.001); and mood (Z = −7.530, *p* < 0.001). Overall, the differences were statistically significant (*p* < 0.05) for all dimensions at 1 month post-intervention and 3 months post-intervention, with the exception of the differences at 2 post-intervention time points for duration and 3 months post-intervention for disease control, which were not statistically significant (Table 5).

**Table 5:** Effects of the nursing intervention on brief illness perception.

Variables	Time period	Intervention group（*n* = 38)	Control group（*n* = 39)	*t/Z*	*P*
BIPQ total scores	T_0_	55.03 ± 2.52	54.31 ± 2.13	1.353^a^	0.180
	T_1_	50.76 ± 2.57	53.69 ± 2.47	−5.095^a^	<0.001
	T_2_	47.89 ± 2.65	52.51 ± 2.37	−8.065^a^	<0.001
Outcome	T_0_	9（8, 9）	8（8, 9）	−1.891^b^	0.059
	T_1_	6（6, 7）	8（7, 9）	−6.400^b^	<0.001
	T_2_	4.5（4, 5）	7（6, 8）	−6.799^b^	<0.001
Duration	T_0_	8（7, 8）	8（7, 8）	−0.253^b^	0.800
	T_1_	7（7, 8）	8（7, 8）	−1.208^b^	0.227
	T_2_	7（7, 8）	8（7, 8）	−1.338^b^	0.181
Personal control	T_0_	4（3.75, 4）	4（4, 4）	−0.515^b^	0.607
	T_1_	6（5, 6.25）	4（4, 5）	−5.579^b^	<0.001
	T_2_	7（6, 7）	5（5, 6）	−6.35^b^	<0.001
Disease control	T_0_	5（4, 6）	5（4, 6）	−0.536^b^	0.592
	T_1_	6（5.75, 7）	5（5, 6）	−2.679^b^	0.007
	T_2_	6（5, 7）	6（5, 6）	−1.421^b^	0.155
Symptoms	T_0_	8（7, 9）	8（8, 8）	−0.295^b^	0.768
	T_1_	6（5, 6.25）	7（7, 8）	−6.17^b^	<0.001
	T_2_	5（4, 5.25）	7（6, 8）	−6.752^b^	<0.001
Attention	T_0_	9（8, 9）	8（8, 9）	−0.701^b^	0.484
	T_1_	7（7, 8）	8（8, 9）	−2.928^b^	0.003
	T_2_	6（5, 6）	5（4, 6）	−3.957^b^	<0.001
Understanding	T_0_	4.5（4, 5）	5（4, 5）	−0.202^b^	0.84
	T_1_	6（5, 6）	5（4, 6）	−3.267^b^	0.001
	T_2_	7（6, 8）	5（4, 6）	−6.469^b^	<0.001
Mood	T_0_	8（8, 9）	8（7, 9）	−1.763^b^	0.078
	T_1_	6（5.75, 7）	8（7, 8）	−5.497^b^	<0.001
	T_2_	4（4, 5）	7（7, 8）	−7.530^b^	<0.001

T0 pre-intervention.

T1 1-month post-intervention.

T2 3-months post-intervention.

^a^ *t*-value.

^b^ *Z*-value.

### 4.5 The effect of the nursing intervention on participants' emotion regulation self-efficacy

At the pre-test, the difference between the control group and the intervention group in terms of total emotion regulation self-efficacy score and scores of each dimension was not statistically significant [t = −1.461, Z = (−1.74) −(−0.681), all *p* > 0.05]. At posttest, the intervention group at T1 had significantly higher total emotion regulation self-efficacy scores and scores on all dimensions than the control group, with total emotion regulation self-efficacy (Z = −4.889, *p* < 0.001); positive (Z = −5.356, *p* < 0.001); and frustration (Z = −3.93, *p* < 0.001); and anger (Z = −3.428, *p* < 0.05).The intervention group of T2 had significantly higher emotion regulation self-efficacy total scores and scores on all dimensions (except exercise monitoring compliance) than the control group, with the EMOTION REGULATION SELF efficacy total score (Z = −7.534, *p* < 0.001); positive (Z = −7.604, *p* < 0.001); frustration (Z = −7.304, *p* < 0.001); and anger (Z = −1.328, *p* > 0.05). Overall, except for the self-efficacy score for regulating angry emotions, for which the difference in data at 3 months post-intervention was not statistically significant, the differences in all other dimensions were statistically significant (*p* < 0.05) at 1 month post-intervention and 3 months post-intervention (Table 6).

**Table 6:** Effects of the nursing intervention on emotion regulation self-efficacy.

Variables	Time period	Intervention group（*n* = 38)	Control group（*n* = 39)	*t/Z*	*P*
Total scores	T_0_	32.71 ± 2.44	33.46 ± 2.06	−1.461^a^	0.148
	T_1_	36.5（35, 38）	34（33, 35）	−4.889^b^	<0.001
	T_2_	41（40, 42）	35（34, 36）	−7.534^b^	<0.001
Positive	T_0_	11（10, 12）	11（10, 12）	−0.681^b^	0.496
	T_1_	13（12, 14）	11（10, 12）	−5.356^b^	<0.001
	T_2_	15（14, 16）	11（11, 12）	−7.604^b^	<0.001
Frustration	T_0_	11（10, 12）	12（11, 13）	−1.305^b^	0.192
	T_1_	13（11, 14）	11（10, 12）	−3.93^b^	<0.001
	T_2_	15（14, 15）	11（11, 12）	−7.304^b^	<0.001
Anger	T_0_	10（9, 12）	11（10, 12）	−1.74^b^	0.082
	T_1_	11（10, 12）	12（11, 13）	−3.428^b^	0.001
	T_2_	11.5（11, 13）	12（12, 13）	−1.328^b^	0.184

T0 pre-intervention.

T1 1-month post-intervention.

T2 3-months post-intervention.

^a^ *t*-value.

^b^ *Z*-value.

### 4.6 The effect of the nursing intervention on participants' BP, BMI, 6MWT

The results showed that the difference in blood pressure was statistically significant when comparing the two groups at 1 month and 3 months after intervention (*P* < 0.05); the body indices of the two groups were analyzed at 1 month and 3 months after intervention, and the results showed that the difference was statistically significant at 3 months after intervention (*P* < 0.05); the 6MWT scores of the two groups of patients were analyzed at 1 month and 3 months after intervention, and the results showed that the difference was statistically significant (*P* < 0.05) (Table 7).

**Table 7:** Effects of the nursing intervention on BP, BMI, 6MWT.

Variables	Time period	Intervention group（*n* = 38)	Control group（*n* = 39)	*t/Z*	*P*
Systolic blood pressure	T_0_	141.5（135.75, 146）	141（138, 146）	−0.112^b^	0.911
	T_1_	132（125.5, 135）	138（134, 143）	−3.534^b^	<0.001
	T_2_	129（126, 132.25）	136（130, 140）	−3.662^b^	<0.001
Diastolic blood pressure	T_0_	91（86.75, 94）	92（88, 94）	−1.162^b^	0.245
	T_1_	82.63 ± 3.844	86.28 ± 6.316	−3.054^a^	0.003
	T_2_	80.18 ± 3.352	82.69 ± 6.798	−2.061^a^	0.044
BMI	T_0_	23.92 ± 1.9	24.19 ± 2.17	−0.576^a^	0.566
	T_1_	23.34 ± 1.95	24.12 ± 2.18	−1.657^a^	0.102
	T_2_	22.79 ± 1.82	23.7 ± 2.15	−2.013^a^	0.048
6MWT	T_0_	362.34 ± 7.68	366.08 ± 9.05	−1.951^a^	0.055
	T_1_	387.84 ± 9.09	377.33 ± 12.72	4.163^a^	<0.001
	T_2_	414.92 ± 9.53	388.82 ± 13.39	9.875^a^	<0.001

T0 pre-intervention.

T1 1-month post-intervention.

T2 3-months post-intervention.

^a^ *t*-value.

^b^ *Z*-value.

## 5 Discussion

This is the first study to evaluate the impact of a nursing intervention based on a common-sense model of self-regulation on compliance to cardiac exercise rehabilitation in Chinese patients with CHD. Results demonstrated that the 12-week intervention significantly improved participants' exercise compliance, emotion regulation self-efficacy, and reduced participants' fear of exercise, brief ill perception. Participants’ blood pressure, BMI, and 6MWT were significantly improved.

### 5.1 Improved exercise compliance

Our findings showed that the intervention significantly increased participants' exercise compliance, aligning with prior studies (34, 35). First, according to the CSM theoretical framework, we facilitated patients' development of accurate illness perceptions and recovery expectations, thereby enhancing rehabilitation engagement. A multimodal intervention protocol—incorporating bedside coaching, rehabilitation room coaching, WeChat, phone calls, text messages, and outpatient reviews—enabled continuous participant communication and progress monitoring. Tailored exercise regimens were implemented based on participants' rehabilitation settings (inpatient/outpatient), participants were encouraged to perform more aerobic and resistance exercises, flexibility exercises, etc., which helped maintain their enthusiasm for exercise. In addition, the use of exercise bracelets and WeChat campaigns to monitor exercise indicators, along with timely adjustments to the program based on the participant's exercise situation helped to promote exercise compliance among the participants (36).

### 5.2 Improved emotion regulation self-efficacy

Our intervention employed a participant-centered approach, focusing on patients' dynamic mood fluctuations and avoiding didactic health education methods. The results demonstrate that this intervention significantly enhanced participants' emotion regulation self-efficacy. We used various forms of emotion regulation (e.g., role modelling, self-expression) to enhance participants' awareness of the role of self-efficacy in emotion regulation, encourage active regulation of maladaptive emotions, and foster recovery motivation (37). Multiple emotion regulation techniques were employed to enhance participants' awareness of self-efficacy in emotion regulation, promote active regulation of maladaptive emotions, and foster recovery motivation. Additionally, patients who demonstrated superior outcomes in prior exercise rehabilitation programs were recruited to share experiences via online platforms, highlighting the role of peer support in bolstering patients' confidence in emotion regulation self-management (38).

### 5.3 Improved blood pressure, BMI, and 6MWT

Our intervention demonstrated efficacy in enhancing systemic blood pressure, body mass index, and 6MWT outcomes, consistent with previous findings (39). Although no statistically significant difference in BMI was observed between the two participant groups at baseline (T_1_), a sustained intervention resulted in a statistically significant intergroup difference at T_2_. Throughout the intervention, nurses disseminated diet and exercise education materials via the WeChat platform, consistently emphasizing that adopting a low-fat diet and regular exercise could improve health indicators—blood pressure, BMI, and 6MWT (40, 41). They regularly monitored and encouraged participants' health behaviors while engaging family members to provide supplementary support. Studies show women with low heart rate variability (HRV) report greater emotion regulation difficulties than men with low HRV. Conversely, women with high HRV report slightly fewer difficulties than all men. Understanding neurophysiological sex differences in emotion regulation may thus aid daily emotion management, enhancing well-being (42). Furthermore, men and women responded similarly to cardiac rehabilitation for most physiological measures. However, men showed greater improvements in maximal oxygen uptake, functional capacity, 6MWT, and handgrip strength (43). This likely results from sex differences in physiology and body composition, giving men better adaptation in some areas. These findings highlight sex differences' importance, necessitating greater attention in cardiac rehabilitation planning. However, our trial aimed to assess overall intervention effects, with analysis focused on intergroup comparisons (intervention vs. control). Future multicenter trials must prioritize this to fully understand sex differences in cardiac rehabilitation.

### 5.4 Reduced fear of exercise

Evidence indicates that the fear of aerobic exercise is most pronounced in cardiovascular disease patients, where perceived disease status amplifies uncertainty regarding physical sensations (44). The intervention was found effective in reducing exercise fear among participants. Patients received detailed explanations on exercise rehabilitation safety, and exercise programs were modified in real-time according to individual profiles to enhance perceived security. Additionally, the intervention reduced illness perception, ultimately decreasing exercise fear—a result aligning with prior research findings (45). Furthermore, family members provided supplementary support that potentially contributed to reducing patients' fear toward movement.

### 5.5 Reduced ill perception

Our intervention effectively reduced participants' illness perception. We assessed participants' level of illness perception to identify negative emotions early, assisting patients in developing positive coping styles to channel and release such emotions. In addition, we implemented measures to stimulate health beliefs (e.g., conducting lectures and distributing educational brochures) and guided positive illness reappraisal to improve illness perception. Many studies have demonstrated that reducing illness perception and motivating patients to adopt positive coping strategies can improve their health behaviors and exercise compliance (45, 46).

### 5.6 Development of telehealth cardiac rehabilitation

Strong evidence supports cardiac rehabilitation (CR) as a clinically effective and cost-effective intervention for most cardiovascular patients. Home-based and technology-supported CR models, increasingly evidenced as alternatives or adjuncts to traditional center-based programs, offer scalable, affordable solutions, particularly in low- and middle-income countries with limited CR access (6). Telehealth CR is a feasible and effective alternative to conventional outpatient CR (47), with demonstrated long-term efficacy in coronary artery disease patients (11). The home- (±digital/telehealth platforms) and centre-based forms of cardiac rehabilitation formally supported by healthcare staff effectively improve clinical and health-related quality of life outcomes in cardiovascular patients (48). Additionally, mobile health solutions enhance patient care and operational efficiency (49).

A Common-Sense Model (CSM)-based nursing intervention significantly improved exercise adherence in coronary heart disease (CHD) patients. This evidence-based approach can be integrated into telehealth CR platforms to overcome access barriers. Incorporating CSM's self-regulation principles—targeting illness perceptions and coping procedures—into remote programs addresses unmet rehabilitation needs in resource-limited settings. Particularly in developing countries with low traditional CR access, telehealth CR utilizing this model provides a scalable approach to expand coverage, reduce costs, and standardize care globally. The intervention's established efficacy offers a framework for enhancing remote CR systems.

## 6 Strengths

This pioneering study is the first globally to apply the CSM to enhance cardiac exercise rehabilitation compliance in CHD patients. Our hospital-led, home-based model bridges hospital and home care, addressing patient barriers like time constraints, distance, and costs, while overcoming hospital limitations such as space, equipment, and staff shortages. Multimodal communication—face-to-face, telephone, and online platforms—diversifies health education and improves patient engagement.

## 7 Limitation

Several limitations exist in our study. The sample size was small and all participants in one city. Thus, the sample were not representative of patients with CHD in other settings or in other regions of China. Should the intervention time be extended, some of the intervention effects could have been different. Moreover, future multicenter trials could explore gender differences in cardiac rehabilitation in depth to enhance the effectiveness of personalized implementation of intervention programs.

## 8 Conclusion

In conclusion, CSM-based nursing interventions can improve CHD cardiac exercise rehabilitation compliance and health outcomes. This pioneering study deploys a hospital-led home-based model that overcomes patient accessibility barriers and institutional resource constraints via multimodal engagement to significantly enhance CHD rehabilitation compliance. This study provides a reference for future enhancement of health education and intervention for patients with CHD. The use of larger representative samples and longer intervention periods should be considered in future studies.

## Data Availability

The datasets are not readily available because this study is an interventional research involving clinical indicators and identifiable health information of patients. The informed consent form explicitly states that the collected data may only be used for the specific purposes of this research project. During the informed consent process, participants were neither informed nor consented to public archiving or sharing of their raw data. Publicly sharing this data would violate the contractual agreement and trust established with participants through the informed consent documentation. Requests to access the datasets should be directed to the corresponding author.
